# Nuclear FOXO3 predicts adverse clinical outcome and promotes tumor angiogenesis in neuroblastoma

**DOI:** 10.18632/oncotarget.12728

**Published:** 2016-10-18

**Authors:** Judith Hagenbuchner, Martina Rupp, Christina Salvador, Bernhard Meister, Ursula Kiechl-Kohlendorfer, Thomas Müller, Kathrin Geiger, Consolato Sergi, Petra Obexer, Michael J. Ausserlechner

**Affiliations:** ^1^ Departments of Pediatrics II, Medical University Innsbruck, Innsbruck, Austria; ^2^ Pediatrics I, Medical University Innsbruck, Innsbruck, Austria; ^3^ Tyrolean Cancer Research Institute, Innsbruck, Austria; ^4^ Walter C. Mackenzie Centre, University of Alberta, Edmonton, Canada

**Keywords:** chorioallantoic membrane (CAM), hypoxia, chemotherapy

## Abstract

Neuroblastoma is the most frequent, extracranial solid tumor in children with still poor prognosis in stage IV disease. In this study, we analyzed FOXO3-phosphorylation and cellular localization in tumor biopsies and determined the function of this homeostasis regulator *in vitro* and *in vivo*. FOXO3-phosphorylation at threonine-32 (T32) and nuclear localization in biopsies significantly correlated with stage IV disease. DNA-damaging drugs induced nuclear accumulation of FOXO3, which was associated with elevated T32-phosphorylation in stage IV-derived neuroblastoma cells, thereby reflecting the *in situ* results. In contrast, hypoxic conditions repressed PKB-activity and caused dephosphorylation of FOXO3 in both, stroma-like SH-EP and high-stage-derived STA-NB15 cells. The activation of an ectopically-expressed FOXO3 in these cells reduced viability at normoxia, but promoted growth at hypoxic conditions and elevated VEGF-C-expression. In chorioallantoic membrane (CAM) assays STA-NB15 tumors with ectopic FOXO3 showed increased micro-vessel formation and, when xenografted into nude mice, a gene-dosage-dependent effect of FOXO3 in high-stage STA-NB15 cells became evident: low-level activation increased tumor-vascularization, whereas hyper-activation repressed tumor growth.

The combined data suggest that, depending on the mode and intensity of activation, cellular FOXO3 acts as a homeostasis regulator promoting tumor growth at hypoxic conditions and tumor angiogenesis in high-stage neuroblastoma.

## INTRODUCTION

Chemotherapy resistance is a hallmark of aggressive neuroblastoma (NB). A resistant phenotype correlates with aberrant expression of brain derived neurotrophic factor (BDNF) and its cognate receptor NTRK2/TrkB [1]. BDNF/TrkB induces survival signaling via the phosphatidylinositol-3-kinase (PI3K) - protein kinase B (PKB) pathway. In addition a retrospective study suggests that phosphorylation of PKB at serine-473 (S473) and/or threonine-308 (T308) correlates with a decreased event-free and overall survival in NB [2] suggesting that PKB might be involved in deregulation of NB growth. Substrates of PKB are the members of the FOXO transcription factor family, FOXO1/FKHR, FOXO3/FKHRL1, FOXO4/AFX and FOXO6 that are involved in various cellular processes ranging from cell death induction to longevity regulation [3]. We and others have shown that FOXO3 is phosphorylated by hyperactive PKB in malignant NB cell lines [1, 4]. The phosphorylation by PKB induces the association of FOXOs with 14-3-3 proteins resulting in their export from the nucleus and repression of their target gene transcription [5-7]. In the absence of PKB-activity FOXOs localize to the nucleus where they control the transcription of genes that are critical for cell death induction, cell cycle progression and detoxification [8, 9]. Oxidative and genotoxic stress induce FOXO-phosphorylation on distinct threonine residues by c-Jun N-terminal kinase (JNK) and mammalian sterile 20-like kinase 1 (MST1), which leads to the translocation to the nucleus even in the presence of growth factor signaling [10-12]. FOXO3 also interacts with ataxia telangiectasia mutated (ATM), which modulates the response to genotoxic stress and the expression of silenced caspase 8 *via* the ATM-target cAMP-responsive element binding protein (CREB) [13, 14]. The opposed functions of FOXOs, i.e. induction of programmed cell death or induction of longevity have been ascribed to posttranslational modification by histone acetyltransferases/deacetylases and to the interaction with various transcription factors [3, 15].

FOXO transcription factors share the same DNA binding motif and seem to have overlapping functions, although knock-out animals for single FOXO family members show different defects: whereas FOXO1 knock-out mice die during embryonic development due to defective vasculature, FOXO3 and FOXO4 knock-out mice show a mild phenotype [16]. However, conditional triple knock-out mice provide evidence that FOXO1, FOXO3, FOXO4 are critically involved in the maintenance of the haematopoietic stem cell population and the regulation of endothelial cell homeostasis [17, 18], whereas FOXO6 depletion protects against fat-induced disorders in mice [19].

Although FOXOs are generally seen as tumor suppressor proteins emerging data also suggest that the FOXO transcription factor FOXO3 may even support cancer development by protecting tumor cells against oxidative stress [12], by promoting drug resistance in cooperation with other FOX transcription factors [20], activating PKB survival signaling [21], attenuating the pro-apoptotic response to hypoxia [22] and even by promoting tumor cell invasion *via* induction of matrix metalloproteinases [23]. Especially its role in autophagy and cellular metabolism [24, 25] may critically influence the survival of tumor cells in solid tumors to overcome hypoxia and nutrition-depletion-induced crisis when the tumor grows above a certain size.

In this study we demonstrate for the first time that nuclear, PKB-phosphorylated FOXO3 expression correlates with high-risk NB and reduced patient survival. We uncovered that low-level activation of FOXO3 promotes cell growth under hypoxic conditions and tumor angiogenesis *in vivo*, whereas strong activation reduces tumor size and leads to deposition of extracellular matrix within the tumor tissue.

## RESULTS

### FOXO3 is localized to the nucleus despite PKB phosphorylation in stage IV NB patients

**Figure 1:**
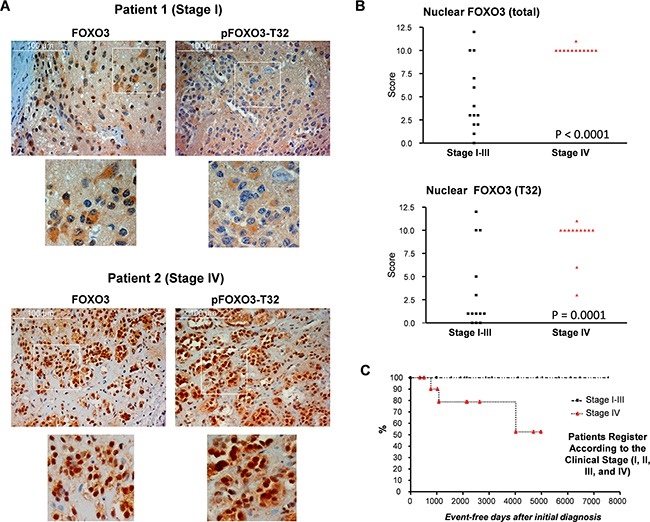
Expression analyses of NB tumor tissue reveals correlation between nuclear, T32-phosphorylated FOXO3, stage IV disease and adverse clinical outcome FOXO3 expression, localization and its phosphorylation at the PKB-site T32 was analyzed in 25 different NB patients by immunohistochemistry. Shown are examples of tissue sections from a low-stage and a high-stage patient **A.** Statistical analyses of immunostainings for FOXO3 and pFOXO3-T32 of NB biopsies **B.** Kaplan-meier survival curves of event-free survival after diagnosis **C.** Statistical analysis was performed using two-way ANOVA.

To explore the potential role of FOXO3 as a disease marker in human NB, we analyzed FOXO3 expression, its subcellular localization and its phosphorylation at the PKB phosphorylation site threonine-32 (pFOXO3-T32) in a set of human NB biopsies. NB samples were scored according to intensity and nuclear localization. The staining of FOXO3 and pFOXO3-T32 revealed both, cytoplasmic and nuclear localization in tumor samples. In Figure 1A exemplary samples of two patients are shown. Patient 1 (Figure 1A, upper panel) who was classified as NB stage I disease, showed a preferentially cytoplasmic staining for FOXO3 and detectable cytoplasmic pFOXO3-T32 staining. In contrast, NB cells from patient 2 (Figure 1A, lower panel, stage IV disease) presented pronounced, almost exclusive nuclear FOXO3 and pFOXO3-T32 staining. Analysis of biopsies from all 25 NB patients revealed a highly significant correlation between stage IV NB and nuclear localization of FOXO3 (P<0.0001) and pFOXO3-T32 (Figure 1B, ANOVA). All stage IV NB biopsies presented nuclear FOXO3 and 83% of these patients also showed pronounced pFOXO3-T32 staining (P=0.0001). We conclude from these data that the subcellular localization of FOXO3 in malignant neuroblasts is independent of PKB activity and nuclear FOXO3 is associated with high-stage NB and reduced event-free patient survival (Figure 1C).

### PKB phosphorylation status of FOXO3 varies between different NB cell lines

Next we analyzed the PKB- and FOXO3-status in a panel of different NB cell lines isolated from tissue biopsies at the St. Anna Children's hospital in Vienna. As a positive control we used CCRF-CEM, an acute lymphoblastic leukemia cell line that carries a PTEN deletion leading to high PKB activation [26].

**Figure 2:**
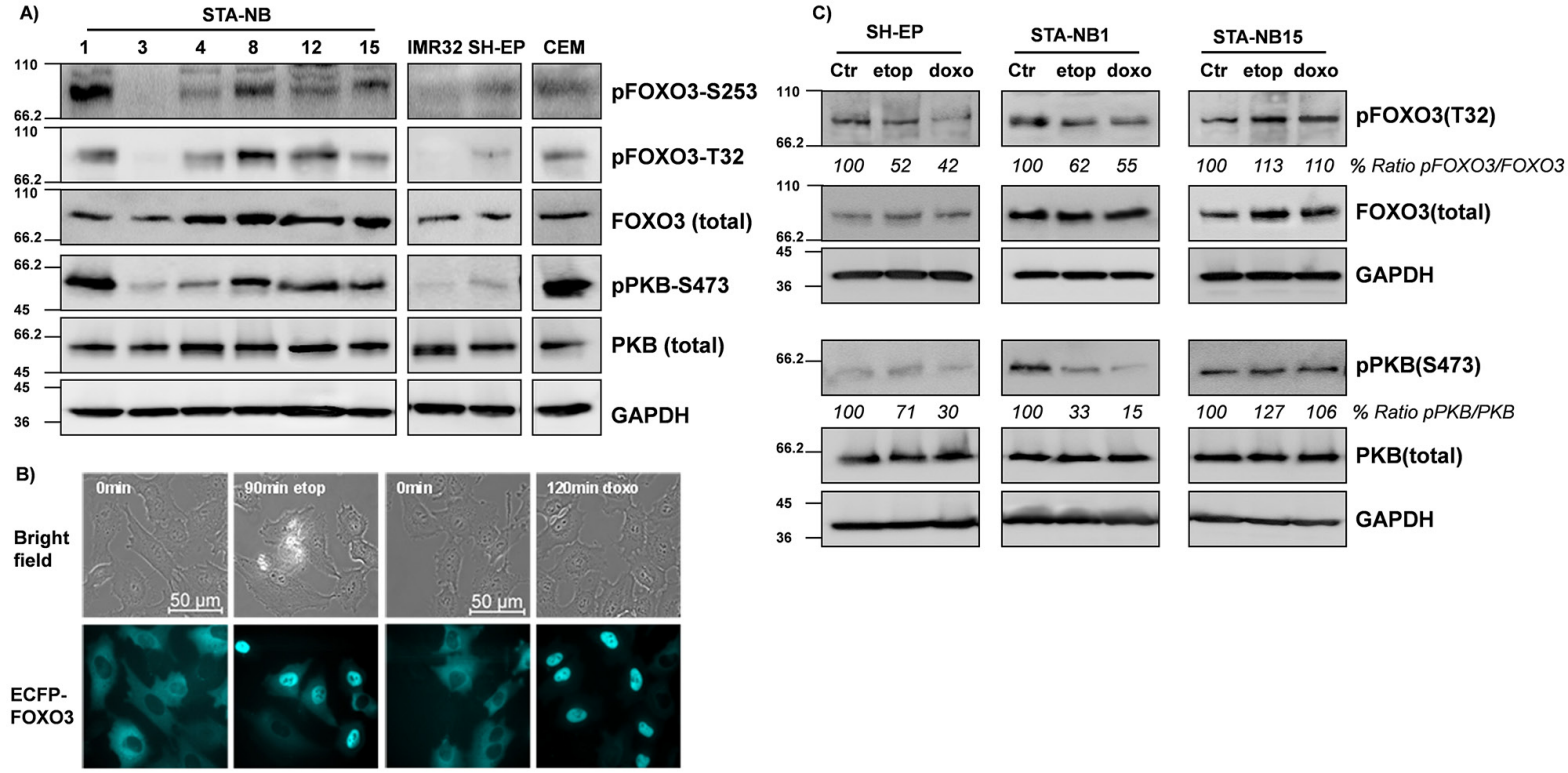
FOXO3 accumulates in the nucleus of NB cells upon drug treatment A panel of patient-derived NB cell lines (STA-NB1, STA-NB3, STA-NB4, STA-NB8, STA-NB12, STA-NB15) and the cell lines SH-EP and IMR32 were analyzed for the expression and phospho-status of FOXO3, PKB, pFOXO3-T32, pFOXO3-S253, and pPKB-S473 by immunoblot. CCRF-CEM leukemia cells were included as a positive control for PKB-hyperactivation **A.** SH-EP/ECFP-FOXO3 cells expressing ECFP-tagged FOXO3 were treated with etoposide (20 μg/ml) for 90 minutes or with doxorubicin (0.25 μg/ml) for 120 minutes and monitored by live cell fluorescence imaging **B.** SH-EP, STA-NB1 and STA-NB15 cells were treated with etoposide (10 μg/ml) and doxorubicin (0.5 μg/ml) for 2 and 6 hours, respectively, and subjected to immunoblot analyses using antibodies directed against FOXO3, pFOXO3-T32, PKB, pPKB-S473 and GAPDH as a housekeeping control **C.**

Significant differences in the expression and activity of the PKB – FOXO3 pathway were observed in cultured cells, ranging from absence of PKB activity to high phosphorylation of FOXO3 at the respective PKB sites threonine-32 (T32) and serine-253 (S253) analyzed (Figure 2A). In relation to the high-stage-derived cell lines the phosphorylation of FOXO3 at the PKB sites T32 and S253 was low in the NB cell lines SH-EP, STA-NB3 and IMR32 and consistently these cell lines also showed moderate to low PKB phosphorylation at the activating site PKB-serine-473 (S473). In contrast, in STA-NB1, STA-NB4, STA-NB8, STA-NB12, and STA-NB15 cells PKB was higher phosphorylated at S473 indicating increased activity of this signaling pathway.

### FOXO3 shuttles to the nucleus upon treatment with DNA-damaging drugs and accumulates in the nucleus despite PKB-phosphorylation

According to the analyses of patient biopsies (Figure 1), nuclear localization and T32-phosphorylation of FOXO3 strongly correlate with stage IV disease, raising the question why PKB-driven export from the nucleus might be hampered in high-stage NB. A possible explanation for this phenomenon might be cellular stress, e.g. hypoxia or DNA-damage that overruns the re-localization signal by PKB phosphorylation. Stress signaling-induced phosphorylation of FOXO3 at serine-209 (S209) by MST1 and 14-3-3 phosphorylation by JNK was reported to interrupt 14-3-3-mediated nuclear export and cause FOXO3 nuclear accumulation despite hyperactivation of PKB [10-12]. However, in NB cells, JNK is neither activated by etoposide nor by doxorubicin and it does not contribute to FOXO3-activation [27]. Instead, we demonstrated that the FOXO3 – ATM – CREB axis [14] is activated in response to genotoxic stress, which causes nuclear accumulation of FOXO3 in drug-treated NB cells [13].

As about 50% of high-stage biopsies were taken after a primary chemotherapy and stage IV NB patients frequently relapse despite high-dose chemotherapy we tested the effect of chemotherapeutic drugs used in NB treatment on FOXO3 expression, phosphorylation and subcellular localization (Figures 2B and 2C and Supplementary Figures S1 and S2).

In a first instance, we used SH-EP cells expressing an ECFP-FOXO3 fusion protein [13] and treated these cells with etoposide (20 μg/ml) or doxorubicin (0.25 μg/ml). In parallel, we also performed immunofluorescence staining of FOXO3 expression to assess the subcellular localization in SH-EP and STA-NB15 cells which we treated with the same chemotherapeutic agents. Both, ectopic ECFP-FOXO3 in SH-EP cells and endogenous FOXO3 accumulated in the nucleus upon drug treatment (Figure 2B and Supplementary Figures S1A and S1B) [27]. Of note, in STA-NB15 cells already in the untreated cell population some cells showed nuclear staining of FOXO3, suggesting partial activation of endogenous FOXO3 also under normal culture conditions.

To investigate, how drug treatment affects FOXO3-phosphorylation and whether some cell lines feature the same FOXO3 phosphorylation phenotype as stage IV NB tumors *in situ*, we analyzed the phosphorylation status of endogenous PKB and FOXO3 in etoposide- and doxorubicin-treated SH-EP, STA-NB1 and STA-NB15 cells. As shown in Figure 2C, in whole-cell lysates including the nuclei of SH-EP and STA-NB1 cells (isolated from a stage III tumor) phosphorylation on FOXO3-T32 was lowered in treated cells. In contrast, STA-NB15 cells which were derived from a stage IV NB tumor FOXO3 steady state protein levels as well as phosphorylation on T32 significantly increased during etoposide and doxorubicin treatment. The same effect was observed when SH-EP and STA-NB15 cells were treated with busulfan (5 μg/ml), ifosfamide (10 μg/ml), vincristine (75 ng/ml) and cisplatin (10 μg/ml) which are drugs that were also used in initial chemotherapy for patients in Figure 1 (Supplementary Figure S2 and Table S1).

Therefore, concerning FOXO3-expression levels, T32-phosphorylation and subcellular localization, drug-treated STA-NB15 cells mimic the phenotype of neuroblasts in tumor tissue from stage IV patients.

### Increased survival and proliferation of stage IV STA-NB15 cells despite reduced activity of the PI3K - PKB pathway and active FOXO3 at hypoxic conditions

**Figure 3:**
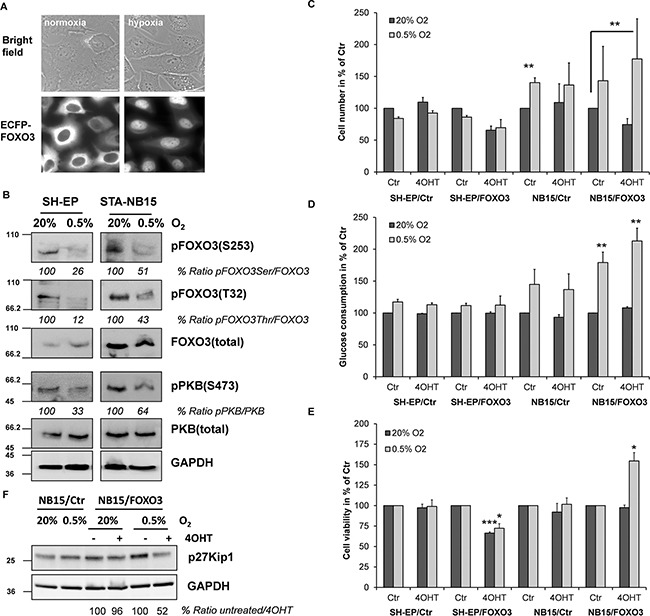
Hypoxia reduces the activity of the PKB survival pathway, causes nuclear accumulation of FOXO3 and promotes growth of high-stage STA-NB15 cells SH-EP/ECFP-FOXO3 cells were cultured for 24 hours at normoxic (20% O_2_) or hypoxic (0.5% O_2_) conditions and then analyzed by live cell fluorescence microscopy **A.** SH-EP and STA-NB15 cells were cultured for 6 hours at normoxic/hypoxic conditions and then subjected to immunoblot analyses with antibodies directed against FOXO3, pFOXO3-T32, pFOXO3-S253, PKB and pPKB-S473. GAPDH served as a housekeeping control **B.** SH-EP/Ctr, SH-EP/FOXO3, NB15/Ctr and NB15/FOXO3 cells were cultured for 24 hours at normoxia or hypoxia in presence or absence of 5 nM 4OHT. The number of viable cells was assessed using a Casy TT cell counter **C.** and alamar blue assay **E.** Glucose content of the cell culture media was measured after 24 hours using a BioVision glucose assay kit **D.** Shown is the mean of three experiments. Statistical significance was assessed with student's t-test (* p < 0.05, ** p < 0.01, *** p < 0.001). Cell pellets of cells cultured at normoxic or hypoxic conditions for 24 hours in presence or absence of 5 nM 4OHT were subjected to immunoblot using antibodies directed against p27^Kip1^ and GAPDH **F.**

Besides drug-induced stress also hypoxic conditions in solid tumors might affect the FOXO3 activation status and such a specific microenvironment might even steer alternative FOXO3 responses. To test this hypothesis we cultured SH-EP/ECFP-FOXO3 cells for 24 hours in low serum media and at hypoxic conditions to mimic nutrient and oxygen restriction in a modular incubator chamber and analyzed them by live cell fluorescence imaging. As demonstrated in Figure 3A, hypoxia (0.5% O_2_) induced accumulation of FOXO3 in the nucleus. To assess initial changes of PKB and FOXO3 expression as well as activity-steering phosphorylation on these proteins, SH-EP and STA-NB15 cells were cultured in normoxia (20% O_2_) or hypoxia (0.5% O_2_) in the hypoxia chamber for 6 hours and subjected to immunoblot analyses. SH-EP cells were derived from SK-N-SH NB cells and represent an epithelial, low-stage phenotype that does not form tumors *in vivo*, whereas STA-NB15 cells efficiently grow to solid tumors in nude mice [28] and thereby still represent the malignant stage IV phenotype. The immunoblot analyses revealed a marked reduction of PKB-S473 phosphorylation in both cell lines and, apparently as a consequence thereof, reduced but not completely diminished phosphorylation of FOXO3 at the PKB sites T32 and S253 (Figure 3B), which was in line with the pronounced nuclear accumulation observed in Figure 3A. Hypoxic conditions therefore reduce the activity of the PKB pathway and activate FOXO3.

Based on these findings we next analyzed, how hypoxia influences growth and metabolism of these cells and if a conditionally-activated FOXO3 allele exerts differential effects on cell survival and metabolism at normoxic or hypoxic conditions. For these experiments we used SH-EP and STA-NB15 cell lines designed to express a 4-hydroxy-tamoxifen (4OHT)-activated FOXO3 allele described before [4, 13]. Identical numbers of cells seeded to cell culture flasks were cultured at normoxic (20% O_2_) or hypoxic (0.5% O_2_) conditions for 24 hours. Cell numbers were measured in a Casy TT cell counter (Figure 3C), glucose consumption was assessed by a colorimetric assay (Figure 3D) and the relative number of viable cells was measured by alamar blue assay (Figure 3E). As shown in Figure 3C hypoxic culture conditions reduced the cell number of SH-EP/Ctr cells to 85% compared to untreated normoxic control cells. Low dose activation of ectopic FOXO3 by 4OHT (5 nM) to mimic activation of endogenous FOXO3 further inhibited cell growth to less than 70% in both, normoxic and hypoxic culture conditions, suggesting that this growth inhibitory effect of FOXO3 in the SH-EP cell model was not influenced by O_2_ availability.

In contrast to SH-EP cells, the cell numbers of NB15/Ctr cells increased at hypoxic conditions to 140% of normoxic NB15/Ctr controls and this phenotype was also observed in NB15/FOXO3 cells. The addition of low dose 4OHT to activate the ectopic FOXO3 allele resulted in a pronounced differential response between normoxia and hypoxia. Whereas at normoxia cell numbers of NB15/FOXO3 cells were reduced to 75% (compared to untreated controls), hypoxic conditions changed the FOXO3-response into the opposite direction: Low-dose FOXO3 activation significantly increased cell numbers compared to NB15/Ctr normoxic controls. Consistent with the increased number of NB15/Ctr cells at 0.5% oxygen also overall glucose consumption significantly increased in NB15/FOXO3 cells compared to normoxic conditions (Figure 3D) and this was also reflected by the measurement of viable cells *via* the alamar blue assays (Figure 3E).

When calculating glucose consumption *per* cell it becomes evident that hypoxic conditions significantly increase glucose consumption in SH-EP/Ctr cells. 4OHT-induced activation of FOXO3 by low-dose 4OHT also significantly boosted glucose consumption *per* cell under normoxic as well as hypoxic conditions, demonstrating a pro-glycolytic effect of FOXO3 in these cells (Supplementary Figure S3). In contrast to SH-EP cells, no increased glucose consumption *per* cell was observed in STA-NB15 cells (Supplementary Figure S3), suggesting that these cells, which already mainly rely on glycolysis at normoxia due to high expression of endogenous Survivin [28, 29], mainly benefit from activation of FOXO3 under hypoxic conditions. Of note, also in absence of 4OHT hypoxic NB15/FOXO3 cells metabolized significantly more glucose than NB15/Ctr cells, although cell numbers were not statistically significant increased. This suggests an increased basal FOXO3 activity due to slight leakiness of the ectopically expressed 4OHT-activated FOXO3-estrogen receptor fusion protein (FOXO3(A3)ERtm) compared to mock-infected controls. Survivin-overexpression by a gain of 17q correlates with stage IV NB and is predictive for an adverse clinical outcome [30, 31]. The activation of FOXO3 in such glycolytic tumors, either *via* genotoxic drug treatment or due to hypoxia may provide a significant growth advantage leading to stage IV cancer cells that resist cancer therapy as demonstrated in Figure 1. In support of changes in cell number and metabolic data, the cell cycle inhibitor p27^Kip1^ accumulates at hypoxia and is suppressed by low dose 4OHT correlating with increased number of metabolically active NB15/FOXO3 cells (Figure 3F). Therefore, under hypoxic conditions, FOXO3 changes its function from a tumor suppressor to a growth-promoting transcription factor in those stage IV NB cells that already mainly rely on aerobic glycolysis as main energy source.

### FOXO3 increases micro-vessel formation of NB onplants in chorioallantoic membrane (CAM) assays and changes steady state expression of VEGF-A and VEGF-C

**Figure 4:**
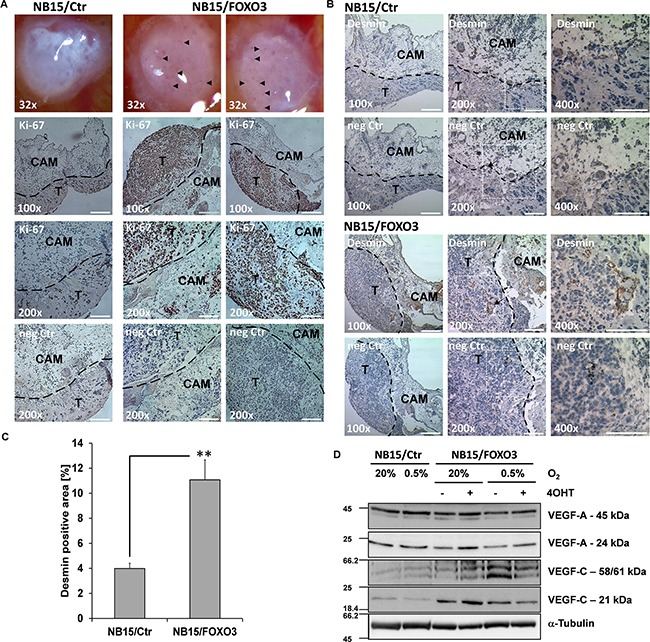
NB15/FOXO3-derived tumors exhibit increased vessel formation in chorioallantoic membrane (CAM) assays NB15/Ctr and NB15/FOXO3 cells were transplanted onto the CAM of 10-days-old chicken embryos and incubated for another five days. Pictures of onplants were taken in a Motic SMZ168 microscope (Motic Deutschland GmbH, Wetzlar, Germany). Microvessels are labelled with an arrow (**A**, top). Paraffin embedded onplants were stained for Ki-67 (proliferating cells, bottom micrographs) and desmin (vessels, **B**) and analyzed in an Axiovert 200M microscope (100x: bars represent 200 μm; 200x: bars represent 100 μm). Desmin-staining of paraffin-embedded onplants was analyzed by densitometry using the Axiovision software. Shown is the mean +SEM of 6 different onplants (micrographs taken at 400x magnification) **C.** Statistical significance was assessed with student's t-test (** p < 0.01). NB15/Ctr and NB15/FOXO3 cells were cultured at normoxic and hypoxic conditions for 24 hours and subjected to immunoblot analyses using antibodies directed against VEGF-A, VEGF-C and GAPDH **D.**

To investigate the effects of ectopically-expressed FOXO3 in an *in vivo* model we next performed CAM assays using NB15/Ctr and NB15/FOXO3 cells as these cells are tumorigenic also *in vivo*. NB15/Ctr and NB15/FOXO3 cells were mixed with collagen and seeded onto the CAM of 10-day-old chicken embryos. Eggs were then incubated for another five days before micro-tumors were photographed and excised for further histological analysis. In Figure 4A, micrographs of CAM onplants derived from NB15/Ctr and NB15/FOXO3 cells are shown: Markedly more NB15/FOXO3 onplants presented with microvessels growing into the onplant compared to NB15/Ctr controls (Figure 4A). Representative CAM-onplants were stained for the proliferation marker Ki-67 to identify the tumor cells (Figure 4A) and the smooth muscle cell marker desmin to stain small vessels (Figure 4B). NB15/FOXO3 cells showed more and clustered proliferating cells and a slightly increased thickness of the onplants. In NB15/FOXO3-derived onplants large, desmin-positive vessels were observed, whereas in CAM-onplants derived from NB15/Ctr cells desmin staining was relatively weak and limited to single cells (Figure 4B). Densitometric analyses revealed an approximately threefold increased desmin-staining in NB15/FOXO3-derived onplants (Figure 4C). To determine whether the increased size and number of micro-vessels in NB15/FOXO3 CAM-onplants reflects hypoxia or FOXO3 effects on the expression of angiogenesis/lymphangiogenesis-modulating cytokines, we analyzed the expression of different variants of VEGF-A (45 kDa and 24 kDa) and VEGF-C (61 kDa, 58 kDa and 21 kDa) under hypoxic conditions and in absence/presence of 5 nM 4OHT to activate the ectopic FOXO3(A3)ER allele (Figure 4C). VEGF proteins undergo complex proteolytic modifications during maturation so that several processed variants of each VEGF member can be detected by western blot. VEGF-A induces growth of blood vessels whereas VEGF-C is implicated in lymphangiogenesis and tumorangiogenesis. Whereas no significant changes in the steady state levels of VEGF-C were induced by hypoxia in NB15/Ctr cells, VEGF-A full length expression slightly increased. Activation of ectopic FOXO3 elevated VEGF-A expression (full length: 45 kDa; processed: 24 kDa) under normoxia and this effect was attenuated at hypoxic conditions. Interestingly, compared to NB15/Ctr cells hypoxic conditions significantly elevated the expression of the VEGF-C variants 61 and 58 kDa in NB15/FOXO3 cells, whereas the processed 21 kDa VEGF-C variant *per se* was higher expressed in NB15/FOXO3 cells than in controls and induced by 4OHT treatment at normoxia. Hypoxic conditions slightly lowered the expression of this variant.

Although these *in vitro* changes in VEGF expression may not fully reflect the *in vivo* situation in a patient or animal, they point towards a gene-dosage-dependent effect of FOXO3 in tumor angiogenesis in NB: On one hand the activation of ectopic FOXO3 induces VEGF-A (24 kD) and on the other hand an apparent low level background activity due to slight leakiness of the ectopic construct in NB15/FOXO3 cells as already demonstrated in Figure 3D, elevates processed (21 kDa) VEGF-C steady state levels at normoxia and increases full length VEGF-C expression at hypoxic conditions. This basal activity of the ectopic FOXO3(A3)ERtm allele in NB15/FOXO3 cells might well mimic the partial activation of endogenous FOXO3 in stage IV patient biopsies as shown in Figure 1. In a final step we therefore transplanted NB15/Ctr and NB15/FOXO3 cells into immune-deficient nude mice and investigated the effect of FOXO3 on tumor growth and tumor vessel formation.

### Gene-dosage determines the effect of FOXO3 on tumor angiogenesis and tumor growth *in vivo*

For *in vivo* experiments, we chose the Balb c nu/nu xenograft mouse model in which we transplanted NB15/Ctr and NB15/FOXO3 tumor cells [28]. Each mouse was injected with 2×10^6^ NB15/Ctr and NB15/FOXO3 cells subcutaneously in the left and right flank, respectively, to directly compare control and FOXO3-expressing cells within each individual. Tumors were grown until palpable and then mice were randomly divided into two groups, one receiving twice a week subcutaneous injections of 4OHT (500μg 4OHT/100μl carrier) to activate ectopic FOXO3, one being injected with carrier only. After three weeks, animals were sacrificed, tumors were measured, photographed and preserved for immunoblot and immunohistological analyses.

**Figure 5:**
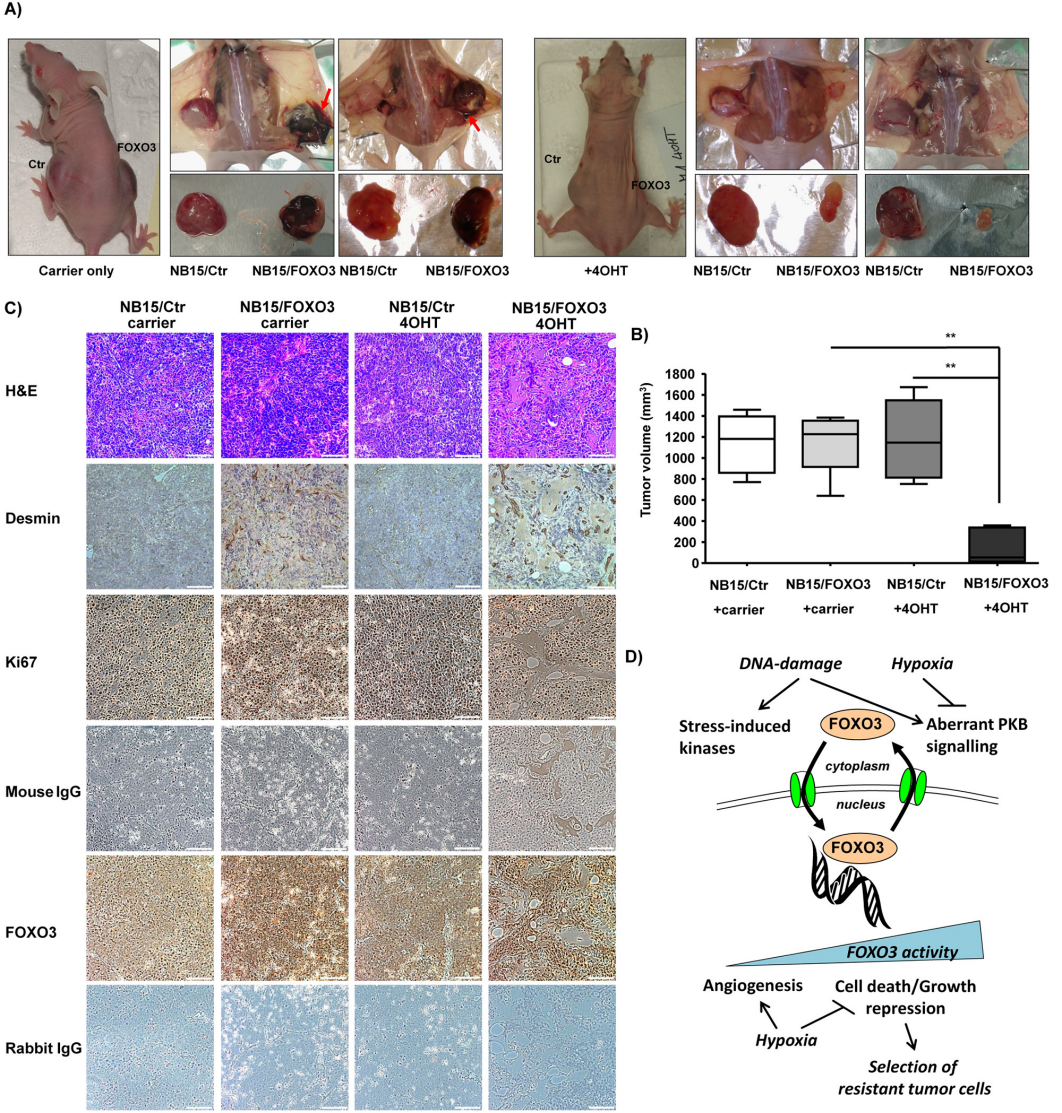
Xenograft transplantation experiments reveal a gene-dosage dependent effect of FOXO3 on NB tumors *in vivo* NB15/Ctr and NB15/FOXO3 cells were injected into the left and the right flank of 6-weeks old Balb c nu/nu mice, respectively, and treatment was started as soon as tumors were palpable (approximately 50 mm^3^). Two times per week 4OHT or carrier-only were injected subcutaneously until control tumors reached a size of more than 1000 mm^3^ (6-8 animals per group; **A**). Animals were sacrificed and tumor size was assessed by an electronic caliper **B.** Tissue was paraffin-embedded, H&E stained and stained for FOXO3, for Ki-67 to detect proliferating cells, and for Desmin to identify micro-vessels within the tumor tissue **C.** *Model for FOXO3 function in high stage NB:* low level FOXO3 activity by genotoxic stress or hypoxia promotes angiogenesis and tumor cell proliferation, whereas strong activation of FOXO3 efficiently represses tumor growth *in vivo*, but in parallel leads to increased cell heterogeneity in the residual tumor tissue that also contains large numbers of tumor cells with high nuclear FOXO3 expression **D.**

Already in living animals a clear difference between tumors developed from NB15/Ctr and NB15/FOXO3 cells was visible (Figure 5A): In carrier-only treated animals the tumors that developed from injected NB15/FOXO3 cells were dark-red suggesting increased vascularization and/or vessel permeability (Figure 5A, left panels). This difference between mock-infected controls (NB15/Ctr) and those expressing ectopic FOXO3 was also evident when tumors were removed (Figure 5A, left panels). Of note, adjacent to several FOXO3 tumors blood-filled subcutaneous plaques were found (Figure 5A, red arrows) which were absent in NB15/Ctr tumors or animals treated with 4OHT. Therefore, low-level FOXO3 activation most likely due to the leakiness of the ectopic construct seems to significantly affect vessel formation and/or vessel permeability. This finding is consistent with the increased vascularization of CAM onplants of NB15/FOXO3 cells and the strong induction of VEGF-C under hypoxic conditions (Figure 4). It suggests that FOXO3-activation in stage IV tumors after chemotherapy, as observed in patient biopsies, may support tumor-vascularization and/or vessel permeablility. In those animals that underwent 4OHT treatment NB15/FOXO3-derived tumors were significantly reduced in tumor size (Figure 5B, P< 0.002), which is consistent with the widely described FOXO3-tumor suppressor function. It underlines a gene-dosage-dependent FOXO3 effect *in vivo*: Slightly increased activity induces tumor-angiogenesis and vessel permeability, whereas strong activation of over-expressed FOXO3 exposes the “FOXO3 tumor suppressor phenotype”.

To study whether FOXO3 gene-dosage affects blood vessel growth into the tumor, we stained NB15/Ctr and NB15/FOXO3-derived tumors for the expression of the smooth muscle cell marker desmin to identify small vessels within the tumor tissue. In addition, we assessed the expression of the proliferation marker Ki-67 and of FOXO3 expression (Figure 5C and at higher magnification in Supplementary Figure S4). An intense desmin staining around numerous small caverns was detected in paraffin-embedded tissue from mock-treated NB15/FOXO3 tumors (Figure 5C). Whereas 4OHT treatment did not affect tumor growth or tissue architecture in NB15/Ctr cells (Figure 5C), the activation of ectopic FOXO3 in NB15/FOXO3 cells not only impaired tumor growth as shown in Figure 5B, but also completely changed the histology of the cancer tissue: A prominent desmin-staining was visible around large vessels and, dispersed throughout the tumor, many desmin-negative caverns and large areas of extracellular matrix deposition with strongly reduced numbers of tumor cells were also detected (Figure 5C, middle, right pictures). This suggests that similar to earlier *in vitro* experiments [4], the hyper-activation of ectopic FOXO3 causes programmed cell death of large numbers of NB cells also *in vivo*, but did not completely eradicate them in the tumors, possibly as a consequence of different oxygen availability in distinct areas of the tumor. However, despite the large vessels within the tumors, they were not dark-red as the carrier-only treated NB15/FOXO3-derived tumors suggesting that tissue color and surrounding blood plaques may be due to increased vessel permeability rather than increased tumor vascularization – a phenomenon that is lost, when FOXO3 is strongly activated.

Staining for the proliferation marker Ki-67 was comparable between NB15/Ctr and untreated NB15/FOXO3-derived tumors (Figure 5C and Supplementary Figure S4), which is consistent with the similar growth kinetics of these tumors *in vivo* (Figure 5B). In 4OHT-treated NB15/FOXO3-derived tumor tissue fewer proliferating cells were present. This suggests that FOXO3-activation significantly changed tumor-tissue architecture and reduced tumor cell numbers, but did not completely eliminate proliferating tumor cells. Therefore, we also analyzed whether there might be a selective process towards deletion of (ectopic) FOXO3 and stained the tumor sections also for FOXO3-expression. As shown in Figure 5C (and Supplementary Figure S4) there is a significant difference in the staining intensity between NB15/Ctr and NB15/FOXO3-derived tumors with nuclear FOXO3-staining also in the absence of 4OHT. Interestingly, also in 4OHT-treated tumors still a large number of neuroblasts with pronounced FOXO3-staining exist. This suggests that despite significant reduction of tumor-size, areas of massive extracellular matrix deposition and deletion of a large number of tumor cells, also a significant number of FOXO3-expressing tumor cells persisted. In parallel, presumably due to selective pressure, also the number of FOXO3-negative tumor cells increased during therapy, suggesting increased heterogeneity of the residual tumor tissue after 4OHT-treatment, similar to stage IV NB tumors. This implies that the micro-environment, e.g. hypoxic and nutrient-deprived areas within the tumor tissue significantly affects the physiologic outcome of FOXO3-activation in NB tumor cells.

## DISCUSSION

FOXO transcription factors have been widely described as tumor suppressor proteins that integrate growth factor- and stress-signaling to control cell cycle progression and cell death. This assumption was demonstrated for various cancer types. Also in NB, we and others provided evidence that FOXO3, as a critical death-inducing transcription factor downstream of the PI3K - PKB survival signaling pathway, regulates cell death in this childhood malignancy [4, 27, 32, 33]. However, the role of FOXO3 in NB development might be more complex as also suggested by the discovery that FOXOs play pivotal roles in stem cell survival and may primarily act as homeostasis regulators (reviewed in [3, 15]. In this respect, FOXO3 promotes cancer stem cell survival [34], metastasis in colon cancer [35] and drug resistance [21, 36], suggesting that tumor cells may “learn” under certain micro-environmental circumstances to exploit the “longevity talent” of FOXO3.

Cellular stress, as it occurs during chemotherapy, hypoxia or nutrient-deprivation in tumor tissue, may trigger such a “longevity program” or select those cells that have already reprogrammed themselves. To gain more insights into FOXO3 function in NB we analyzed subcellular localization and phosphorylation of FOXO3 at the PKB phosphorylation site T32 in a collection of tumor biopsies from pediatric NB patients. According to data on PKB activity from Opel et al [2] and more recent work from Santo et al [33] we expected phosphorylated and cytoplasmic FOXO3 in high-stage NB. However, in contrast to our hypothesis, the analysis of biopsies from NB patients revealed a highly significant correlation (P<0.0001, ANOVA) between nuclear FOXO3, stage IV disease and poor patient survival (Figure 1C). The same was also true when the phosphorylation status of FOXO3 at the PKB phosphorylation site T32 was included into the analyses – again nuclear pFOXO3-T32 was highly significant for stage IV disease (P<0.0001, ANOVA) and reduced event-free patient survival (Figure 1C). These results are consistent with studies from Opel et al [2] showing that high PKB activity correlates with adverse outcome. However, as phosphorylation of FOXO3 at conserved PKB sites (T32, S253, S315) should induce the association of nuclear FOXO3 with 14-3-3 proteins and the export of this complex into the cytoplasm, this finding was somewhat surprising and suggested that similar to studies on invasive ductal breast carcinoma [37] the PKB - FOXO3 axis may be disrupted in high-stage NB. One explanation is that chemotherapy, hypoxia and/or nutrient-deprivation during tumor growth constitute stress signals that cause nuclear accumulation of FOXO3 also in cells with a highly active PKB pathway. In this respect stress signaling-induced phosphorylation of FOXO3 at serine-S209 (S209) by MST1 and 14-3-3 phosphorylation by JNK was reported to interrupt 14-3-3-mediated nuclear export and cause FOXO3 nuclear accumulation despite hyper-activation of PKB [10-12]. However, in NB cells, JNK is neither activated by etoposide nor by doxorubicin and it does not contribute to FOXO3-activation [27]. Genotoxic stress activates the FOXO3-ATM-CREB axis [14], which causes nuclear accumulation of FOXO3 in drug-treated NB cells [13].

In a panel of different NB cell lines marked differences in PKB and FOXO3-phosphorylation were observed with pPKB-S473, pFOXO3-T32, pFOXO-S253 being absent or moderate in some cell lines (STA-NB3, SH-EP, IMR32) and mostly more pronounced in high-stage-derived NB cells (STA-NB1, STA-NB4, STA-NB8, STA-NB12, STA-NB15) (Figure 2A). This resembles the heterogeneity of NB tumors *in vivo* and suggests that cultured patient-derived cancer cells reflect the stage-dependent differences in PKB-activity and FOXO3-phosphorylation observed in tissue biopsies (Figure 1). We therefore investigated the subcellular localization of FOXO3, PKB-activity and FOXO3-phosphorylation during treatment with genotoxic drugs. Subcellular localization was assessed by live cell imaging using SH-EP cells that express an ECFP-tagged FOXO3 fusion protein and by immunofluorescence. In SH-EP cells FOXO3 is almost completely localized to the cytoplasm and treatment with etoposide and doxorubicin caused rapid nuclear accumulation within 120 minutes (Figure 2B), supporting similar observations from earlier studies [13, 27, 38]. Immunofluorescence staining demonstrated that in stage IV STA-NB15 cells endogenous FOXO3 partially localizes to the nucleus also in untreated cells (Supplementary Figure S1). Interestingly, the response of the PKB - FOXO3 signaling pathway to chemotherapeutic drugs used in the therapy of NB strongly differed between the three chosen cell lines SH-EP which represents a stromal phenotype, STA-NB1 (stage III) and STA-NB15 (stage IV): whereas in SH-EP and STA-NB1 cells FOXO3 phosphorylation was reduced during drug-treatment, in STA-NB15 cells PKB-signaling remained constant and FOXO3 steady state levels as well as phosphorylation strongly increased (Figure 2C). The current therapy for high stage NB is based on a cocktail of chemotherapeutic agents including also cisplatin and cyclophosphamides besides doxorubicin and etoposide. As demonstrated in Supplementary Figure S2, a differential response of SH-EP and STA-NB15 cells was also observed with busulfan, cisplatin, ifosfamide and vincristine. As FOXO3 is more stable in the nucleus than in the cytoplasm the accumulation is a further indirect evidence of FOXO3 activation despite high PKB activity in these cells. Therefore, in high-stage-derived NB cells FOXO3-inactivation by PKB is disrupted by an unknown mechanism, possibly *via* stress-induced kinases [11, 12]. During chemotherapy, hypoxia or nutrient deprivation in the rapidly growing tumor, the majority of tumor cells undergo cell death possibly in part due to strong activation of FOXO3 [4, 27] and in aggressive tumors a selection for aberrant PKB-activation occurs [2]. However, residual NB cells may tolerate or even benefit from FOXO3 activation in presence of hyperactive PKB, which will contribute to the development of more aggressive tumor cells and disease relapse. The selection for survival might be due to genetic/epigenetic alterations of specific tumor cells [39] and a specific, possibly hypoxic or nutrient-deprived micro-environment, which changes the physiologic outcome of FOXO3 activation. In colon carcinoma the interaction of FOXO3 with β-catenin confers resistance to FOXO3-induced cell death and instead leads to a highly metastatic phenotype [35]. As hypoxia also caused FOXO3-activation (Figure 3A), we studied how a hypoxic, low-nutrient environment, as it is present in solid tumors, affects growth and metabolism of stromal-like SH-EP and stage IV-derived STA-NB15 cells. Interestingly, the two NB cell lines showed a different response to these conditions, with STA-NB15 cells exhibiting increased proliferation at hypoxia, whereas proliferation of SH-EP cells decreased (Figure 3C). In contrast to normoxia, where FOXO3-activation reduced growth of both cell types, hypoxic conditions inverted the FOXO3-response in STA-NB15 cells leading to an increased number of viable, metabolically-active tumor cells. Consistent with higher cell numbers, also the overall glucose consumption increased in STA-NB15 cells at hypoxic conditions (Figure 3D). We recently demonstrated that Survivin, a member of the inhibitor of apoptosis protein family that is frequently overexpressed by gain of 17q in high-stage NB, reprograms the metabolism of this tumor and induces aerobic glycolysis [9, 28, 29]. STA-NB15 cells carry a gain of 17q and therefore mainly rely on glycolysis as main energy producing pathway. Low oxygen availability therefore does not critically hamper their way of energy production. Apparently, reduced oxygen levels even improve the growth of these cells, possibly due to reduced ROS levels that otherwise impair viability [27] and/or the inhibition of hypoxia-induced cell death by FOXO3 [22]. Of note, hypoxia significantly reduced PKB-activity and FOXO3-phosphorylation (Figure 3B), which did not impair growth in STA-NB15 cells (Figure 3C), suggesting that FOXO3 activation during hypoxia does not cause cell cycle arrest or cell death in these cells. These results may also explain discrepancies to the data of a recent paper by Santo et al [33] who demonstrated that FOXO3 merely acts as a tumor suppressor and is inactivated in high-stage NB cells. Of note, Santo et al only analyzed gene expression profiling data of NB samples and all *in vitro* experiments presented in that paper were done at normoxic conditions which, according to our data, do not completely reflect the *in vivo* conditions in solid tumors. SH-EP cells, in contrast, showed the expected adaptation to hypoxia with reduction of cell growth (Figure 3C and 3E) and elevated glucose consumption *per* cell (Supplementary Figure S3). Increased glycolysis by FOXO3 is in line with reports that FOXO3-mediated repression of c-Myc reduces mitochondrial DNA copy number and shuts down mitochondrial gene expression and respiration [40, 41]. The combined data suggest that at hypoxic conditions FOXO3 reduces viability of SH-EP cells, whereas in high-stage derived, highly glycolytic STA-NB15 cells, FOXO3 even promotes survival. Since the expression of ectopic FOXO3 already increased metabolic activity of NB15/FOXO3 cells during hypoxia in absence of the FOXO3-activating drug 4OHT, we took advantage of this slightly increased basal FOXO3 activity as it might better reflect the *in vivo* situation in stage IV patients than full-activation of the ectopically expressed FOXO3.

In CAM assays a larger number of micro-vessels was observed in onplants derived from NB15/FOXO3 cells (Figure 4A) and this was also reflected on a histological level by increased vessel formation as detected by threefold-increased desmin-staining (Figure 4B and 4C). We therefore next analyzed whether NB15/FOXO3 cells might produce more angiogenesis-promoting factors than NB15/Ctr cells. Although in breast cancer, a repressive effect of FOXO3 on VEGF was reported [42], we observed that NB15/FOXO3 cells *per se* expressed higher levels of fully processed VEGF-C and hypoxia further induced the unprocessed VEGF-C variant at 58/61 kDa. VEGF-C was described as an inducer of lymphangiogenesis *via* its binding to the VEGF-R3 receptor, but it can also regulate growth and the permeability of blood vessels. The fully processed form of VEGF-C (21 kDa) has a high affinity to both, the VEGF-R3 and VEGF-R2 and was implicated as a critical factor for tumor angiogenesis [43], which might be relevant for the observed angiogenic effects in CAM onplants (Figure 4A and 4B). The increased VEGF-C expression (21 kDa) in NB15/FOXO3 cells also correlates with the dark-red appearance observed in NB15/FOXO3-derived carrier only-treated tumors and the blood-filled plaques surrounding tumor tissue (Figure 5A). These xenograft mouse transplantation experiments finally clearly demonstrated the “Janus-faced” nature of FOXO3 in NB: NB15/FOXO3 tumors in carrier-only treated animals exhibited significant vascularization and/or vessel permeability whereas 4OHT-treated animals had small tumors with few cancer cells (Figures 5A and 5C). Of note, 4OHT-treatment was started when tumors were just palpable at a tumor size when diffusion is sufficient to prevent hypoxic areas within the tumor and therefore FOXO3 mostly acted growth-repressive similar to NB15/FOXO3 cells cultured at normoxia (Figure 3C). In addition, the dose of 500 μg 4OHT/mouse may cause full activation of ectopic FOXO3, thereby better exposing the “tumor suppressor phenotype” as also observed *in vitro* [4]. In such tumors a massive deposition of ECM and significant loss of tumor cells leading to cell-free caverns was observed, suggesting that during FOXO3-induced cell death also fibrosis-inducing factors may be released from neuroblasts (Figure 5C). However, the activation of FOXO3 did not fully eradicate proliferating tumor cells, but promoted heterogeneity of the residual tumor tissue with some FOXO3-negative tumor cells and NB cells with high, nuclear FOXO3 levels that apparently persisted during 4OHT-induced FOXO3 activation (Figure 5C and Supplementary Figure S4). Further studies will show whether these resisting cells exhibit a changed drug-resistance or metastatic phenotype. These observations point towards a critical involvement of micro-environmental factors such as hypoxia or nutrient-deprivation in steering cell fate after FOXO3-activation and questions FOXO3 as a “typical tumor suppressor protein” in NB therapy. From the combined data we therefore conclude that FOXO3 in stage IV NB induces tumor vascularization and thereby rather supports tumor growth and metastasis than acts as a tumor suppressor.

So how do our results relate to previous reports on FOXO3 as a tumor suppressor in NB? Santo et al demonstrated that low FOXO3 activity correlates with poor prognostic outcome [33] as he observed the repression of FOXO3 target genes in high-stage NB. However, this publication is based on mRNA gene expression profiling data and experimental data was based on SH-SY5Y cells cultured at normoxic conditions, similar to our own previous studies. Therefore, they did not address the impact of hypoxia or nutrient-deprivation in fast growing, high-stage NB tumors, which apparently completely changes the response to FOXO3 activation in stage IV NB cells. Our data on NB tumors support other studies from e.g. leukemia and breast cancer, where the FOXO3 “death program” was perverted into a “tumor longevity program” with increased death resistance and invasion [23, 44]. An additional level of complexity is added by the hypoxic and nutrient-deprived conditions within solid tumors that apparently affect physiological outcome of FOXO3 activation [22] in high-stage NB. This “Janus-faced” nature was clearly demonstrated by the FOXO3 effect on tumor-angiogenesis in xenograft animals, suggesting a model (Figure 5D) where low-level activation drives tumor-angiogenesis and the full activation of the ectopic FOXO3 efficiently represses tumor growth, but leads to the selection of resistant tumor cells. We conclude from our study that in high-stage NB patients, FOXO3 contributes to tumor cell longevity associated with death resistance and increased tumor vascularization.

## MATERIALS AND METHODS

### Cell lines and culture conditions

The patient-derived MYCN-amplified NB cell lines STA-NB1, STA-NB3, STA-NB4, STA-NB8, STA-NB12 and STA-NB15 were isolated at the St. Anna Children's Hospital, Vienna [45, 46] and the IMR32 cell line was purchased from ATCC. Phoenix™ packaging cells for helper-free production of amphotropic retroviruses were cultured as previously described, CCRF-CEM leukemia cells, retrovirally infected SH-EP and STA-NB15 cells were described before [4, 27, 47,48].

### Hypoxia experiments and glucose measurements

Cells were plated in 25 cm^2^ cell culture flasks and grown to 80% confluency before they were transferred into 0.5% FCS containing HEPES-buffered medium and incubated in a modular incubator chamber (Billups-Rothenberg, San Diego, CA, USA) at 0.5% oxygen for the times indicated. Cell numbers were measured in a Casy TT cell counter (Roche-Diagnostics, Indianapolis, USA). Glucose content of cell culture media was assessed by a colorimetric method using a commercial Glucose assay kit (BioVision, Mountain View, CA, USA) and measured in a microplate reader (Hidex Chameleon, Turku, Finnland) according to the manufacturer's instruction.

### Viability analysis

Viability of cells was determined with the alamar blue assay (AbD Serotec, UK) in a microplatereader (Hidex, Turku, Finnland) according to manufacturer's instructions.

### Immunohistochemistry of patient biopsies

Formalin-fixed and paraffin-embedded tissue sections of 25 NB patients treated at the Department of Pediatrics, University Hospital Innsbruck from 1989 to 2008 were used for *in situ* staining of FOXO3 (Supplementary Table S1). Written informed consent for the scientific use of paraffin-embedded material was obtained. Sections were stained using the streptavidin-biotin-peroxidase method (Vectastain, Burlingame, Ca. USA) with antibodies specific for FOXO3 and pFOXO3-T32. Briefly, 3 μm thick sections were deparaffinated and rehydrated. Slides were then boiled in a microwave oven in a plastic jar filled with citrate acid buffer solution, pH 6, for 15 minutes. The endogenous peroxidase activity was blocked using a hydrogen peroxide solution. Tissue sections were then incubated with normal bovine serum (10%) for 20 minutes to reduce nonspecific background staining. Primary antibodies specific for FOXO3 (Upstate Biotechnology, CA, USA) and pFOXO3-T32 (Upstate Biotechnology, CA, USA) were applied to tissue sections for 60 minutes at room temperature followed by incubation with a polyvalent antibody for 30 minutes and then an avidin-biotin-complex reagent (Vectastain, Vector Laboratories, UK) for 30 minutes at room temperature. In negative controls, sections were not incubated with primary antibody. Diaminobenzidine tetrahydrochloride was used for staining. All slides were counterstained with hematoxylin, dehydrated, and mounted. Immunostaining results were evaluated, graded, and scored by extent (0 = none, 1 = 1–25%, 2 = 26–50%, 3 = 51–75%, 4 = 76–100% of the ductal cells) and intensity (0 = negative, 1 = weak, 2 = moderate, and 3 = intense). An immunohistochemical score was calculated for each case in which the percentage of positive rating was multiplied by the intensity rating. All specimens were centrally evaluated and their diagnosis was confirmed by the National Registry of NB, Vienna, Austria. To avoid artifacts, only well formalin-fixed tissue specimens (buffered 4% formalin) with expression of NB markers were studied. The pathologist screening the samples was blinded to clinical and survival data and further experimental results.

### Immunoblotting

Immunoblot analysis was performed as described previously [48] using primary antibodies directed against human FOXO3, pFOXO3-T32, pFOXO3-S253, PKB and pPKB-S473 (Cell Signaling Technology, Boston, USA), GAPDH (Acris antibody GmbH, Herford, Germany), VEGF-A, p27Kip1 (BD-Austria, Vienna, Austria), VEGF-C (Thermofisher Scientific, Waltham, USA) and α-Tubulin (Calbiochem, SanDiego, CA, USA). Afterwards the membranes were washed and incubated with horseradish-peroxidase-conjugated anti-mouse or anti-rabbit secondary antibodies (Amersham Biosciences, Buckinghamshire, UK). The blots were developed using enhanced chemiluminescence substrate and quantified in AutoChemi detection system. Densitometry analysis was performed using LabWorks software (UVP, Cambridge, UK).

### Chorioallantoic membrane (CAM) assays and immunohistochemistry

Fertilized chicken eggs were incubated for three days in an 80% humidified 38 °C egg-incubator (Grumbach, Germany) to allow normal embryo development. The experimental procedure is largely based on a protocol by Deryugina et al [49]. On day three of embryonal development, 5 to 7 ml of egg white was removed and a 2×2 cm window was cut into the eggshell, sealed and the eggs were incubated for another seven days. On day 10, when CAM and its vasculature were well developed 2×10^6^ NB15/Ctr and NB15/FOXO3 cells were transplanted. The cells were resuspended in a 50 μl drop of ice-cold collagen dissolved in cell culture medium and the mixture was solidified for 30 minutes at 37 °C. One to two onplants per egg were grafted onto the CAM. After sealing, the living embryos were incubated for another 5 days. Afterwards the embryos were sacrificed by hypothermia. The size and vascularization was documented by microscopy before onplants were excised and paraffin-embedded for further histological analysis. Proliferating cells were identified with an antibody directed against Ki-67 (Dako-Austria, Vienna), smooth muscle cells around micro-vessels were stained using an anti-desmin antibody (Dako-Austria, Vienna).

### Xenograft transplantation experiments

Six-week-old female nude mice (BALB c nu/nu) were purchased from Charles River, Germany. The mice were maintained in an SPF animal facility of the Medical University Innsbruck. Animals were housed with standard 12 h light/dark cycles with food and water *ad libitum*. The experiments were performed according to the guidelines of the Austrian “Tierversuchsgesetz” and approved by the local animal ethics committee (BMBWK-66.011/0011-BrGT/2007; Austria). 2×10^6^ NB15/Ctr and NB15/FOXO3 cells were mixed with 100 μl Matrigel™ (BD Biosciences, San Jose, USA) in PBS (50/50) and injected subcutaneously into the left and right flank, respectively. When the tumors reached a palpable size of approximately 50 mm^3^, animals were injected twice a week subcutaneously with 4OHT dissolved in sterile sunflower oil (500 μg/100 μl). Control groups received sunflower oil without 4OHT. After three weeks animals were sacrificed by cervical dislocation, size of the tumors was measured using a caliper and tumor tissue was paraffin-embedded for further histological analysis. Statistical differences between the groups were assessed by Mann Whitney U test using Graphpad Prism 4.0 software. Immunohistochemistry on paraffin-embedded tissue was done as for the analysis of onplant tissue from CAM assays. FOXO3 was stained using an anti-FOXO3 antibody from Cell Signaling Technology (Boston, USA).

## SUPPLEMENTARY MATERIALS FIGURES AND TABLE




