# Supplementary material for: Nuclear FOXO3 predicts adverse clinical outcome and promotes tumor angiogenesis in neuroblastoma
Source: Oncotarget. 2016 Oct 18;7(47):77591–606. doi: 10.18632/oncotarget.12728 (PMC5363607; doi:10.18632/oncotarget.12728)
Supplement: Supplementary file 1 [file oncotarget-07-77591-s001.pdf]

# Nuclear FOXO3 predicts adverse clinical outcome and promotes tumor angiogenesis in neuroblastoma

## SUPPLEMENTARY FIGURES AND TABLE

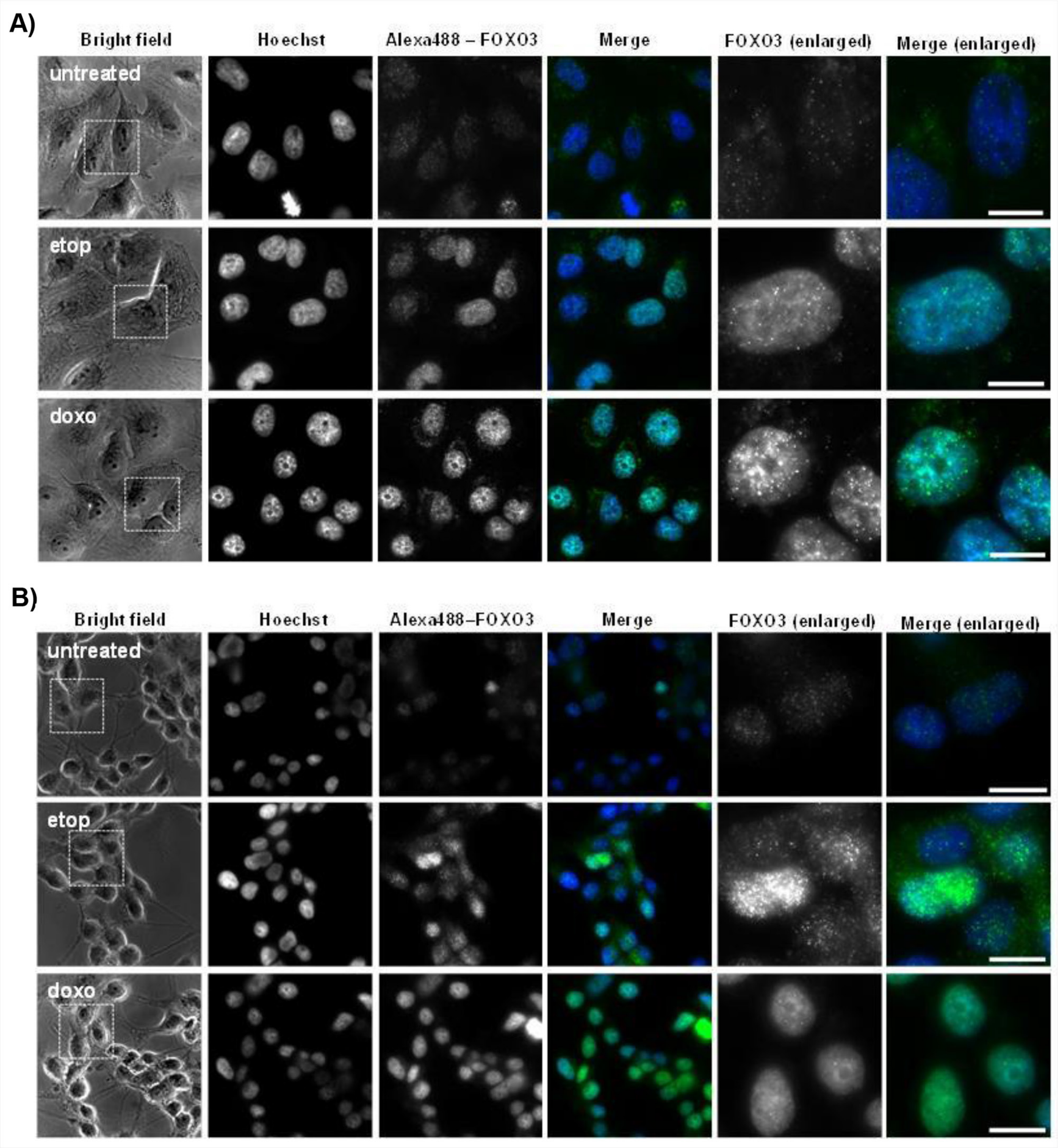


**Supplementary Figure S1: FOXO3 accumulates in the nucleus of NB cells during drug treatment.** SH-EP cells **A.** were treated with 20 µg/ml etoposide or 0.25 µg/ml doxorubicin for 3 hours. STA-NB15 cells **B.** were treated with etoposide (10 µg/ml) or doxorubicin (0.5 µg/ml) for 3 hours. Cells were fixed in 4% paraformaldehyd, permeabilized with 0.1% TritonX-100, blocked with BSA and then subjected to immunofluorescence staining with Alexa488-labeled antibodies directed against human FOXO3. DNA was stained using Hoechst 33342. Images were acquired on an Axiovert 200M microscope and analyzed by Axiovision software (Zeiss, Vienna). The staining intensity was enhanced for SH-EP cells compared to STA-NB15 cells to make the FOXO3 signal visible.

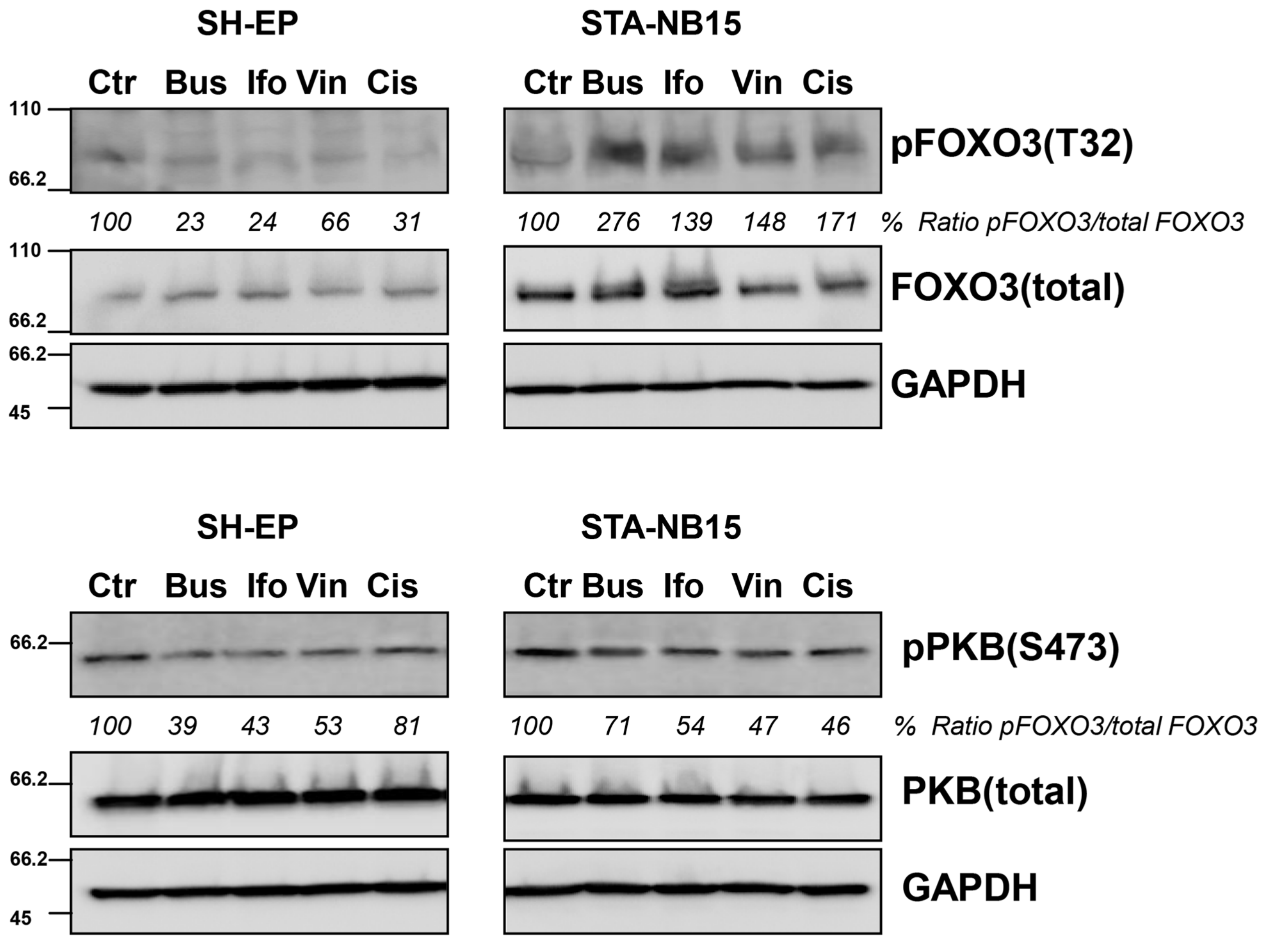


**Supplementary Figure S2: Effects of busulfan, ifosfamide, vincristine or cisplatin on FOXO3- and PKB-steady state expression and phosphorylation.** SH-EP, and STA-NB15 cells were treated with 5 µg/ml busulfan, 10 µg/ml ifosfamide, 75 ng/ml vincristine or 10 µg/ml cisplatin for 3 hours and subjected to immunoblot analyses using antibodies directed against FOXO3, pFOXO3-T32, PKB, pPKB-S473 and GAPDH as a housekeeping control. Densitometry analysis was performed using LabWorks software (UVP, Cambridge, UK).

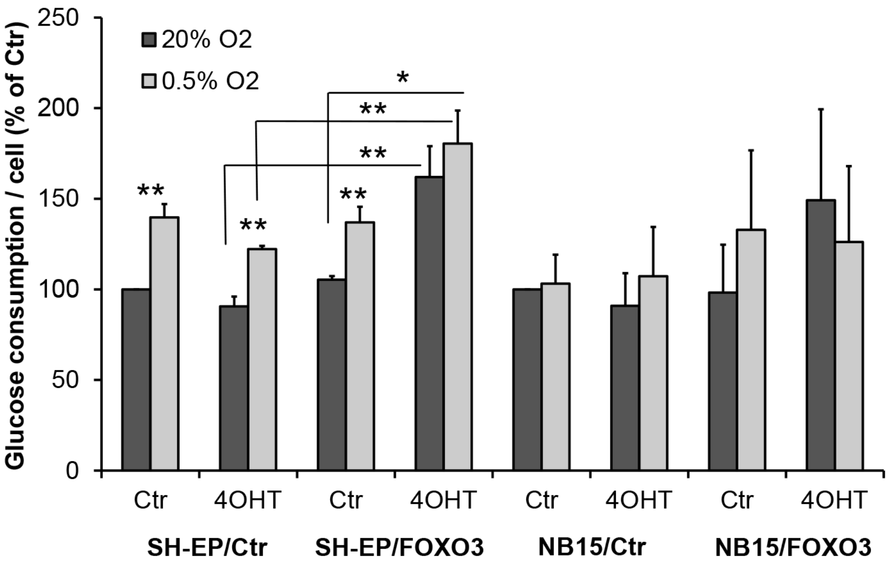


**Supplementary Figure S3: Differences in glucose consumption *per cell* between SH-EP and STA-NB15 cells.** SH-EP/Ctr, SH-EP/FOXO3, NB15/Ctr and NB15/FOXO3 cells were cultured for 24 hours at normoxia or hypoxia in presence or absence of 5 nM 4OHT. Glucose content of the cell culture media was measured after 24 hours using a BioVision glucose assay kit. Shown is the mean of three independent experiments. Statistical significance was assessed with student's t-test (* $p < 0.05$, ** $p < 0.01$).

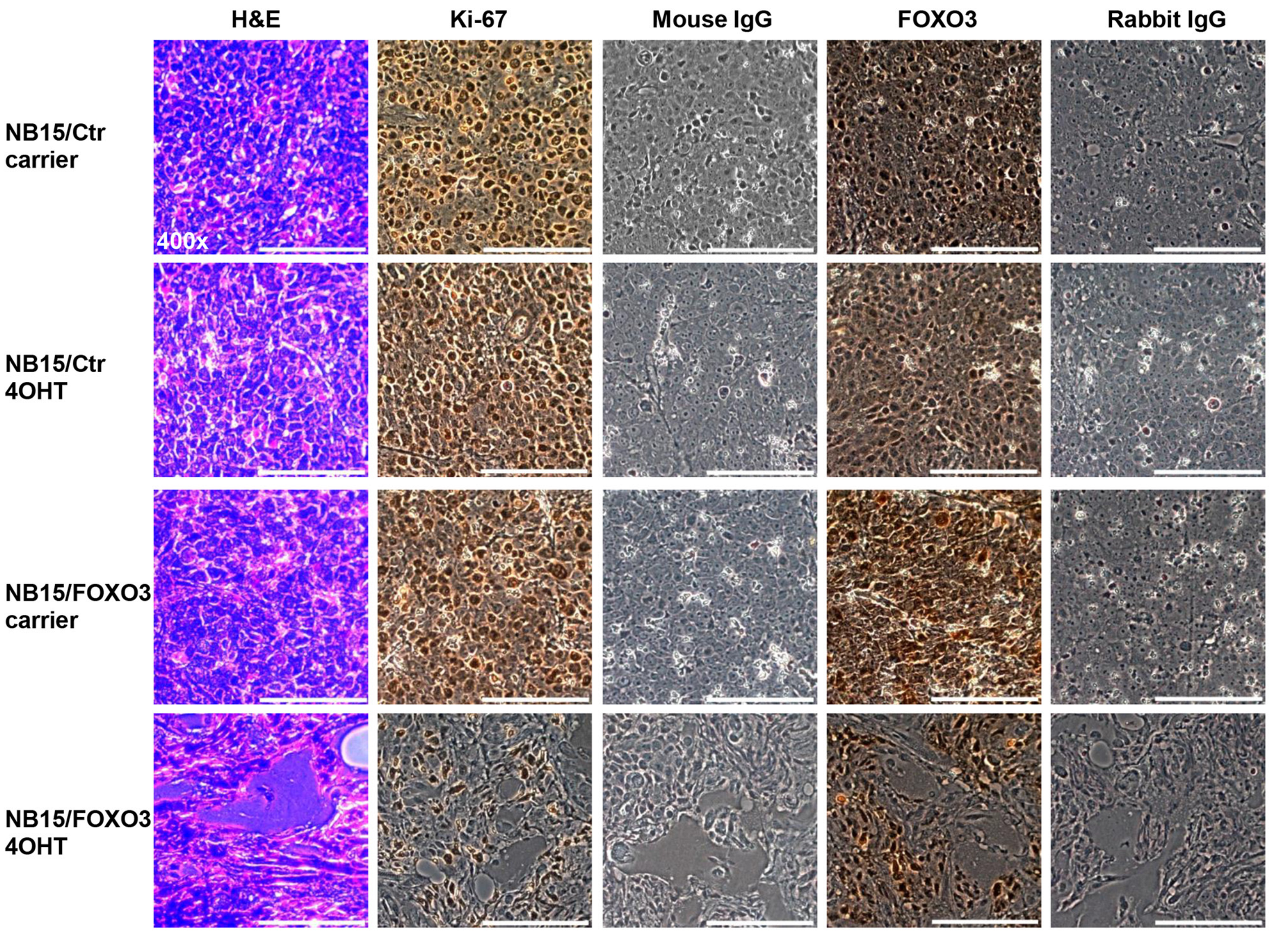


**Supplementary Figure S4: Ki67 and FOXO3 expression in xenografted mouse tumors.** Randomly selected, paraffin-embedded tumors were stained for Ki-67 (proliferating cells) and FOXO3 and analyzed in an Axiovert 200M microscope (400 fold magnification).

**Supplementary Table S1: Neuroblastoma Samples Innsbruck.**

**See Supplementary File 1**
